# Para-aortic lymph node metastases in pancreatic cancer should not be considered a watershed for curative resection

**DOI:** 10.1038/s41598-017-08165-w

**Published:** 2017-08-09

**Authors:** Sebastian Hempel, Verena Plodeck, Franz Mierke, Marius Distler, Daniela E. Aust, Hans-Detlev Saeger, Jürgen Weitz, Thilo Welsch

**Affiliations:** 1 Department of Visceral, Thoracic and Vascular Surgery, University Hospital Carl Gustav Carus, TU Dresden, Dresden, Germany; 2 Department of Diagnostic and Interventional Radiology, University Hospital Carl Gustav Carus, TU Dresden, Dresden, Germany; 3 Institute for Pathology, University Hospital Carl Gustav Carus, TU Dresden, Dresden, Germany

## Abstract

No international consensus regarding the resection of the para-aortic lymph node (PALN) station Ln16b1 during pancreatoduodenectomy for pancreatic ductal adenocarcinoma (PDAC) has been reached. The present retrospectively investigated 264 patients with PDAC who underwent curative pancreatoduodenectomy or total pancreatectomy between 2005–2015. In 95 cases, the PALN were separately labelled and histopathologically analysed. Metastatic PALN (PALN+) were found in 14.7% (14/95). PALN+ stage was associated with increased regional lymph node metastasis. The median overall survival (OS) of patients with metastatic PALN and with non-metastatic PALN (PALN−) was 14.1 and 20.2 months, respectively. Five of the PALN+ patients (36%) survived >19 months. The OS of PALN+ and those staged pN1 PALN− was not significantly different (P = 0.743). Patients who underwent surgical exploration or palliative surgery (n = 194) had a lower median survival of 8.8 (95% confidence interval: 7.3–10.1) months. PALN status could not be reliably predicted by preoperative computed tomography. We concluded that the survival data of PALN+ cases is comparable with advanced pN+ stages; one-third of the patients may expect longer survival after radical resection. Therefore, routine refusal of curative resection in the case of PALN metastasis is not indicated.

## Introduction

One of the unsolved difficulties in treating patients with pancreatic ductal adenocarcinoma (PDAC) is early lymph node spread, which probably leads to tumour recurrence even after complete surgical resection. Consequently, extended lymphadenectomy during pancreatoduodenectomy for PDAC has been studied in multiple trials, which have failed to show results of longer survival^1, 2^. Recently, an International Study Group for Pancreatic Surgery (ISGPS) consensus statement defined the international standard lymphadenectomy based on a current literature review^3^. However, no consensus was reached for recommending routine resection of the para-aortic lymph node (PALN) station Ln16b1 dorsal to the pancreas. Metastasis to this lymph node station is classified as pM1 stage, because these lymph nodes do not belong to the regional lymph node stations. Several studies concluded that PALN metastases are associated with poor survival, but the effect of Ln16b1 metastasis and its resection on survival remained unclear. An updated meta-analysis recently recapitulated the prognostic impact of PALN metastasis and encouraged intraoperative assessment of PALN status. Nevertheless, a clear recommendation whether to complete or to avoid tumour resection in the case of PALN metastasis was not given^4^. In fact, single studies advocated against performing tumour resection if PALN metastasis is histologically verified, thus making PALN metastasis a watershed for curative resection^5^. These contradictive management recommendations warrant further data acquisition and analysis. Almost all available data investigating the impact of PALN resection emanate from Asian – most frequently Japanese – centres. The present study provides additional evidence and aims to assess the prognosis of PALN metastases and their resection in a European population, with a special focus on regional lymph node status.

## Results

### Description of patient, histopathology and morbidity variables

Some 264 patients underwent pancreatoduodenectomy or total pancreatectomy for PDAC within the study period. In 95 cases, the resected PALN were separately labelled and histopathologically analysed. The median follow-up time was 17.9 months. Metastatic PALN (PALN+) were found in 14.7% (14/95) and a mean number of 6 PALN were resected. Most frequently, a pylorus-preserving pancreatoduodenectomy (PPPD) was performed (87%) and most standard patient and surgical characteristics were not significantly different between cases with metastatic and non-metastatic PALN (PALN−) (Table 1). However, the percentage of locally advanced tumours was higher in the PALN+ subgroup, and there was a trend that the PALN+ patients had higher preoperative serum CA 19-9 levels. Moreover, the 14 PALN+ cases had a significant higher rate of regional lymph node spread (N1, 100%; P = 0.002) and a higher lymph node ratio (LNR), reflecting an advanced lymphovascular propagation (Table 2). In the majority of the 14 PALN+ cases, only one PALN was affected (8/14; range of metastatic PALN: 1–7). Postoperative morbidity was equal in both subgroups, except for a significant higher occurrence of postoperative diarrhoea in the PALN+ subgroup. A postoperative pancreatic fistula (POPF) occurred in 21% (PALN+) and 14% (PALN−) (Table 3). The overall 30-day mortality was 2.1% (2/95).

**Table 1:** Patient and operative characteristics.

Variable	PALN+	PALN−	P
Patients [n]	14	81	
Median age [years] (IQR)	68 (61–75)	69 (64–75)	0.236
Male sex [n (%)]	6 (43)	40 (49)	0.775
Diabetes [n (%)]	2 (17)	33 (41)	0.074
Weight loss [n (%)]	9 (64)	40 (49)	0.389
Median CA 19-9 [U/m] (IQR)	202 (47–940)	111 (23–422)	0.060
Resectability status^1^ [n (%])			
Resectable	3/9 (33)	26/44 (59)	0.525
Borderline resectable	3/9 (33)	17/44 (37)	1.000
Unresectable	3/9 (33)	1/44 (2)	**0.026**
Operation [n (%)]			
PPPD	12 (86)	71 (88)	1.000
cPD	1 (7)	3 (4)	0.477
TP	1 (7)	7 (9)	1.000
Neoadj. therapy [n (%)]^2^	3 (21)	11 (14)	0.428
Portal vein resection [n (%)]	9 (64)	39 (48)	0.386
Median operative time [min] (IQR)	390 (315–476)	391 (315–491)	0.664
Adjuvant therapy [n (%)]	12 (86)	66 (81)	1.000

^1^The resectability status was assessed according to the National Comprehensive Cancer Network (NCCN) guidelines and was based on preoperative computed tomography scans (if available).

^2^Neoadjuvant treatment regimen included chemoradiation or chemotherapy according to the FOLFIRINOX protocol; the latter being the current standard protocol in the neoadjuvant setting.Abbreviations: CA 19-9, carbohydrate antigen 19-9; cPD, classic pancreatoduodenectomy; IQR, interquartile range; neoadj., neoadjuvant; PPPD, pylorus-preserving pancreaticoduodenectomy; TP, total pancreatectomy.

**Table 2:** Histopathological tumour characteristics.

Variable	PALN+	PALN−	P
Patients [n]	14	81	—
Tumour grade [n (%)]			
G1/G2	5 (36)	36(44)	0.771
G3/G4	6 (43)	38 (47)	1.000
Gx	3 (21)	7 (9)	0.163
T			
T1/T2	1 (7)	3 (4)	0.477
T3/T4	13 (93)	78 (96)	0.477
N			
N0	0 (0)	37 (46)	**0.001**
N1	14 (100)	44 (55)	
M			
M0	0	81 (100)	
M1	14 (100)	0	**0.001**
Resection status			
R0	8 (57)	62 (77)	0.189
R1	4 (29)	13 (16)	0.269
Rx	2 (14)	6 (7)	0.335
V status			
V0	7 (50)	60 (74)	0.174
V1	6 (43)	19 (23)	
Tumour size [mm] (IQR)	37 (30–40)	33 (27–36)	0.49
Median number of resected LN (IQR)	18 (13–24)	18 (14–24)	0.791
Median number of resected PALN (IQR)	5 (3–8)	3 (2–5)	0.177
LN ratio [n (%)]			
>0,2	6 (43)	18 (22)	**0.019**
≤0,2	8 (57)	63 (78)	
PALN ratio [n (%)]			
>0,2	3 (21)	0	**0.003**
≤0,2	11 (79)	81 (100)	

Abbreviations: IQR, interquartile range; LN, lymph node; PALN, para-aortic lymph nodes.

**Table 3:** Operative morbitity and mortality.

Variable	PALN+	PALN−	P
Patients [n]	14	81	
Morbidity [n (%)]	10 (71)	47 (58)	0.392
Diarrhoea	5 (56)	2 (6)	**0.001**
Lymph fistula	2 (14)	4 (5)	0.214
POPF	3 (21)	11 (14)	0.428
PPH	1 (7)	5 (6)	1.000
DGE	1 (7)	26 (32)	0.062
SSI	2 (14)	15 (19)	1.000
Abscess	2 (14)	6 (7)	0.335
Mortality (30-day) [n (%)]	0	2 (3)	1.000

Abbreviations: POPF, postoperative pancreatic fistulae B/C (classified according to the ISGPS definition from 2005); PPH, postpancreatectomy haemorrhage (ISGPS definition); DGE, delayed gastric emptying (ISGPS definition); SSI, surgical site infection.

### Correlation of PALN status with survival

**Figure 1:**
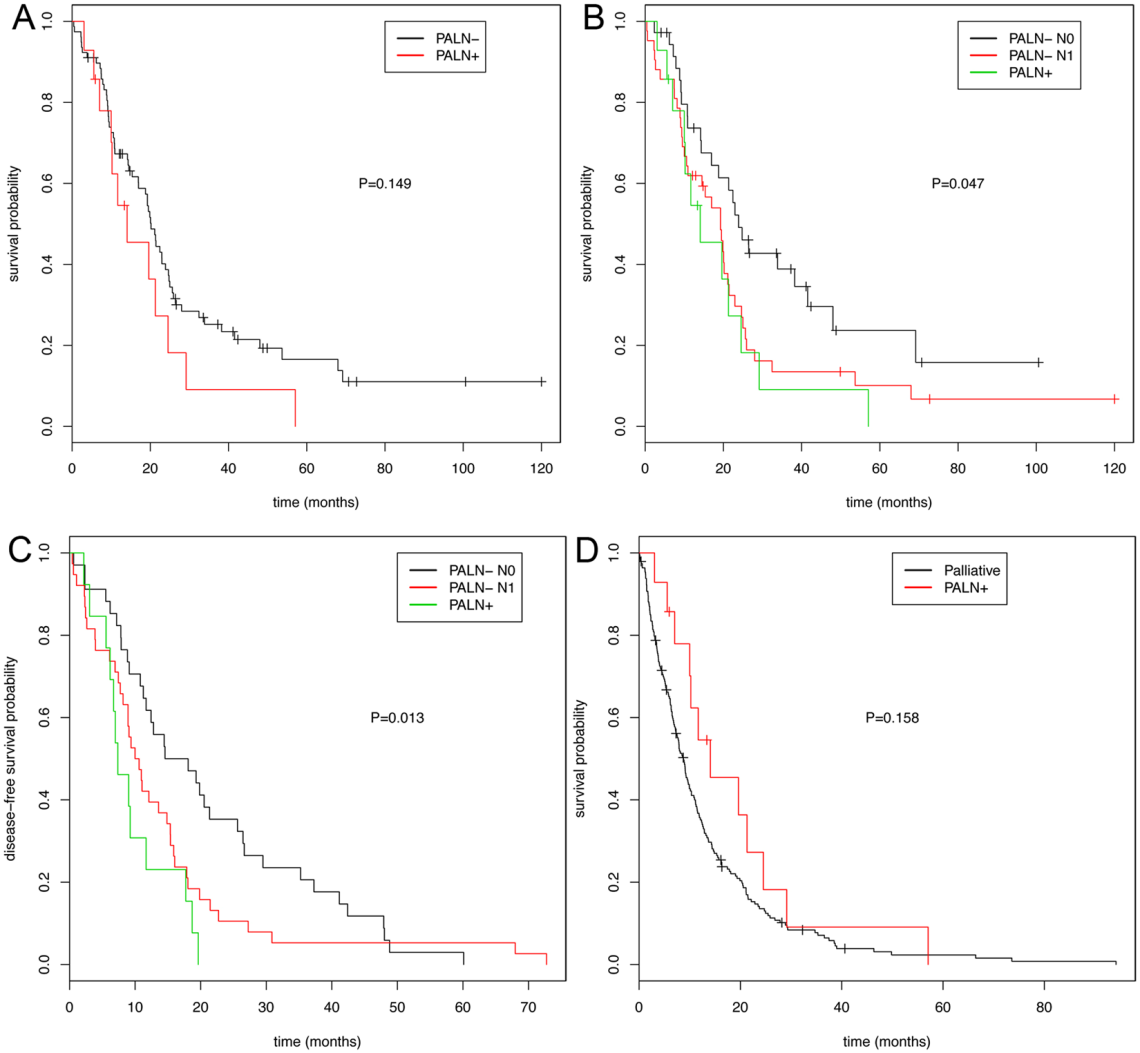
Overall and disease-free survival of patients with para-aortic lymph node resection. (**A**) Overall survival of patients with para-aortic lymph node (PALN) resection (n = 95). Patient subgroups with PALN metastasis (PALN+, n = 14) and without PALN metastasis (PALN−, n = 81) were plotted. (**B**,**C**) Overall survival (**B**) and disease-free survival (**C**) of patients with PALN resection separated into PALN+ (n = 14), PALN− pN0 (n = 37), and PALN− pN1 (n = 44) subgroups according to the regional lymph node status. (**D**) Overall survival of PALN + patients (n = 14) compared with patients after palliative surgery for unresectable or metastastic pancreatic cancer (n = 194).

The median overall/ disease-free survival (DFS) rates of patients with metastatic PALN and with non-metastatic PALN was 14.1 / 7.4 months and 20.2 (95% confidence interval [CI]: 17.0–24.8)/ 12.3 (95% CI 10.0–16.0) months, respectively (Fig. 1). On univariate analysis, PALN status did not significantly correlate with the different overall survival data (P = 0.093). However, PALN status (P = 0.03) and failure to receive adjuvant treatment (P < 0.001) were the only significant factors associated with a shorter DFS on multivariate analysis. To compare para-aortic with regional lymph node metastases, we further stratified cases with non-metastatic PALN according to the pN-stage. The PALN− subgroup included 37 cases staged N0 and 44 cases with regional lymph node metastasis (N1). The median overall survival of the N0 subgroup was 23.9 (95% CI 18.8–48.0) months and significantly longer (P = 0.047), compared with N1 (median survival 19.3 months, 95% CI 10.9–22.9) or PALN+ cases (Fig. 1B). The overall survival of patients with PALN+ and those staged PALN− N1 was not significantly different (P = 0.743). Likewise, the PALN− N0 subgroup had a significant longer DFS (P = 0.013, Fig. 1C).

The new (8^th^ edition) classification of TNM staging of the Union for International Cancer Control (UICC) introduced a N2 stage for PDAC (≥4 regional lymph node metastases) because recent studies had underlined that the number of lymph node metastases significantly affected survival^6, 7^. When we retrospectively applied the 8^th^ edition nodal staging system to the present cohort of patients, the median survival of the patients staged pN0, pN1, and pN2 was 23.9 months, 19.3 months, and 17.0 months, respectively. The pN2 subgroup (n = 24) included 8 patients with PALN metastases, and exclusion of these cases resulted in a median survival of 10.9 months (95% CI 9.4–28.0). In contrast, patients with 1–3 regional lymph node metastases (pN1 according to the 8^th^ edition of TNM staging) and no PALN metastases (n = 28) had a median survival of 19.5 months (95% CI 15.4–25.0). Thus, the survival prognosis of the analysed PALN+ patients was in-between the prognosis of pN1 and pN2 staged cases.

To compare the survival probability of patients with metastatic PALN and palliatively treated patients, we further identified all patients who underwent surgical exploration or palliative bypass surgery for unresectable or metastatic PDAC within the study period. These 194 patients had a median survival of 8.8 (95% CI 7.3–10.1) months, which was not significantly shorter compared with the PALN+ subgroup (Fig. 1D). However, some patients with metastatic PALN demonstrated a long-term survival, not seen in the palliative setting: 5 patients (36%) with positive PALN survived more than 19 months (range 19.6–57.1), mirroring the median outcome of patients treated in curative intent.

### Preoperative prediction of PALN status using computed tomography

Next, it was assessed whether the presence of metastatic PALN could be predicted based on the preoperative CT-imaging studies. The preoperative CT studies were available in only 10 of the 14 patients with positive PALN status. The 10 cases with PALN+ status and available preoperative CT studies were then matched according to age, sex, and tumour stage with 10 PALN− cases (and available preoperative CT studies), and analysed by an experienced radiologist, who was blinded with respect to the histopathological outcome. Positive PALN status was radiologically suspected in only 2 of the 10 cases with PALN metastases (sensitivity: 20%, specificity: 100%) (Table 4). Interestingly, the 2 cases had a high number of tumour-involved PALN (3 and 7), whereas the mean number of metastatic PALN was 1 in non-suspected (based on CT imaging) PALN (Table 4). ^18^F-Fluorodeoxyglucose positron emission/computed tomography (FDG/PET-CT) scans were not performed routinely in the preoperative setting of patients with PDAC. A preoperative FDG/PET-CT was available for only 1 patient with PALN metastases, but the uptake values did not predict the PALN involvement in this case.

**Table 4:** Radiologic prediction of para-aortic lymph node status.

Variable	PALN−	PALN+
Patients [n]	10	10
Tumor stage T3N1 [n]	8	8
Median age [years]	67.8	68.4
Male sex [%]	30	30
Preop. CT available [n]	10	10
Median delay CT to OR [days]	22.5	23.5
Suspected PALN+ [n]	0	2 (20%)
Mean number of pos. LN within the PALN group (non-suspected/suspected PALN)	0	1/5

Abbreviations: CT, computed-tomography; LN, lymph nodes; OR, operation; PALN, para-aortic lymph nodes; preop, preoperative.

## Discussion

The discussion of extended lymphadenectomy or PALN resection during PDAC resection started years ago, but is still an ongoing and controversial debate as demonstrated by the very recent literature. Consequently, no consensus has been reached on the management of PALN^3^. The present study and recent meta-analyses congruently demonstrate that approximately 14–18% of PDAC of the pancreatic head harbour PALN metastases at the time of resection^4, 8^, and that this subgroup of patients has a relative poor prognosis with a median overall survival of 13–14 months. Recently, Hackert *et al*. published an overall median survival of 12.3 months after resection of synchronous oligometastatic PDAC (either liver or interaortocaval lymph node metastases)^9^. Taken together, these data further support that the Asian and European outcome data after PALN resection are comparable. These include the quality of PALN resection, indicated by the mean number of resected PALN: 6 (present study) compared with 4.3^10^ and 5.0^11^ in the Asian studies. Interestingly, our analysis showed that one-third of the patients with PALN metastases survived 19 months or longer after resection. This is in line with results from the largest multicentre study to date, which reported that 26 out of 102 patients with metastatic PALN survived for more than 2 years^10^. There are even reports of long-term survival after removal of PALN metastases^12^. When we compared the median survival duration of patients with palliative surgery (8.8 months) or PALN+ resection (14.1 months), patients in the latter subgroup demonstrated a longer survival of approximately 5 months, thus, supporting the resection of PALN (Ln16b1) at least in subgroups of patients. Based on the fact that PALN metastases are associated with early tumour recurrence, others have recommended that intraoperative sampling of PALN with frozen section analysis should be routinely performed, and consequently, that tumour resection in the case of such lymph node metastases should be avoided^5^. In contrast, we do not conclude that PALN metastases should be considered a general contraindication for curative resection or multimodal strategies, because of the lack of superior treatment alternatives. Moreover, the present data revealed that most frequently only one single lymph node of the Ln16b1 group was tumour infiltrated, which limits the prediction of intraoperative frozen section of single nodes. In future, the possibility of genetic subtype analysis may further define patients who would benefit from PALN resection^13^.

The survival and DFS difference of patients with PALN metastases and regional pN1pM0 stage is marginal, and PALN+ cases are associated with a high burden of lymphovascular tumour spread. The survival prognosis is comparable to the new N2 stage of the UICC 8^th^ edition staging system, which should be evaluated in larger series. Therefore, PALN metastases may characterise a subgroup of PDAC with extensive lymph node metastases, which might benefit additionally from neoadjuvant treatment. The data further open the discussion whether PALN should be classified as regional lymph nodes for PDAC.

However, the preoperative prediction of PALN status is difficult, if not impossible^14^. Imai *et al*. could not detect PALN metastasis in any of their patients by preoperative imaging studies^15^. In the present study, only 2 cases with extensive PALN metastasis (3 and 7 Ln16b1 nodes infiltrated) were identified by the preoperative CT scans. Thus, in most cases the sensitivity of CT imaging is insufficient for prediction of PALN metastases. Other imaging modalities such as FDG/PET-CT or endoscopic ultrasound (EUS) have been investigated in staging of PDAC. Whereas FDG/PET-CT may be more sensitive in the diagnosis of primary PDAC and distant metastases compared with contrast-enhanced CT, its accuracy for detection of local nodal tumor metastases is poor and was reported between 37.5 and 42%^16, 17^. The accuracy of EUS for staging of nodal involvement reached 65% or even higher in single studies, but a meta-analysis demonstrated its general limits in nodal staging based on the pooled accuracy parameters^18, 19^. In summary, the accuracy of CT, FDG/PET-CT or EUS is insufficient to support selective treatment approaches, e.g. neoadjuvant therapy in case of FDG/PET-CT positive PALN.

The present study has some limitations that should be considered. Because PALN resection was routinely performed at our institution, there was no comparison of the morbidity of cases with and without PALN resection. One of the most relevant complications is the development of a POPF, which was diagnosed in 14.7% of all cases. We do not think that dissection of the PALN generally increases the risk for POPF because it does not affect the pancreatic neck dissection or the site of pancreatojejunostomy. A definite allocation of the resected lymph nodes to the Ln16b1 station (PALN) was only possible in 95 of the 264 cases, because PALN were not labelled routinely at the time of resection, which limited the available cases. Further, the low number of cases included for analysis of CT prediction of PALN status is insufficient to calculate valid test statistics, but was predetermined by the 10 available CT studies of patients with PALN metastases.

In conclusion, the present study confirms that patients with PALN metastases (pM1 LYM) have a relative poor prognosis, resulting in a median survival of approximately 14 months. However, one-third of these patients demonstrated a longer survival. Compared with other studies on PALN resection, the present study explicitly analysed the lymph node status and demonstrated that the survival of patients with positive PALN was comparable to patients with pN1pM0 disease, and even more to pN2pM0 patients according to the 8^th^ edition of TNM staging (UICC). Because preoperative imaging studies fail to reliably predict PALN metastases, PALN status prior to tumour resection can only be assessed by intraoperative frozen section analysis. However, abandoning tumour resection in the case of Ln16b1 metastases should not be routinely recommended; we would rather recommend a tailored approach taking into account the individual risk profile of the patient.

## Methods

### Study design and patients

Data were obtained from a prospective database and retrospectively analysed. All patients who underwent partial pylorus-preserving PD (PPPD), classic PD (cPD), or total pancreatectomy (TP) for PDAC of the pancreatic head or neck between January 2005 and March 2015 at the Department of Visceral, Thoracic and Vascular Surgery, University Hospital Carl Gustav Carus, Technische Universität Dresden, Germany, were screened for inclusion. Although PALN resection was performed routinely, the resected lymph nodes could not be unequivocally allocated to the PALN in all cases during pathological workup. Only cases with unequivocally labelled PALN were included for further analysis. The patients were divided into two subgroups: patients with a PALN-positive status (histopathological tumour invasion of at least one PALN, PALN+) and patients without PALN metastasis (PALN−). For survival analysis, PALN-negative cases were subdivided according to the pN stage. Patients who underwent palliative surgery for unresectable or metastatic PDAC in the same observation period were likewise considered for comparative survival analysis.

Tumour recurrence was assessed by regular follow-up examinations in the outpatient clinic, telephone interviews, or by contacting the respective primary physician of the patients. Tumour progression was assumed, if imaging modalities, e.g. sonography, computed tomography (CT) or magnetic resonance imaging (MRI) scans, or the clinical and laboratory examination, e.g. carbohydrate angigen 19-9 (CA 19-9) were indicative. Borderline resectability of the tumours was assessed using the National Comprehensive Cancer Network (NCCN) definition based on the preoperative CT scans^20^. The experimental protocol of the study was approved by the local Ethics Committee of the TU Dresden (decision number EK70022017). All methods were carried out in accordance with relevant guidelines and informed consent was obtained from the all included patients.

### Operative technique

The basic operative technique of the PPPD and cPD was recently described^21^. After an extended Kocher manoeuvre, the PALN were dissected on the ventral aspect and between the vena cava and aorta, beginning at the right side of the coeliac trunk and origin of the superior mesenteric artery to the origin of the inferior mesenteric artery.

### Pathological assessment

Para-aortic lymph node samples were examined macroscopically, dissected and all palpable nodes were submitted for histological examination. Histological analysis was carried out using haematoxylin & eosin slides without serial sectioning or immunohistochemical analyses for single tumour cells. A metastasis was diagnosed when tumour infiltration with desmoplastic reaction was visible.

### Radiological assessment and patient matching

Radiological prediction of postoperative, histopathological PALN tumour invasion was investigated using preoperative CT imaging studies. Therefore, the 14 patients with PALN metastases were matched 1:1 to the patients within the PALN− subgroup, taking into consideration the following variables: age, body mass index (BMI), TNM stage, type of operation. An experienced radiologist, who was blinded with respect to the histopathological outcome, retrospectively analysed the available preoperative CT studies of the cases for assessment of PALN status. The para-aortic anatomical region of the Ln16b1 station was examined on venous-phase sequences. Lymph nodes >1 cm and those showing an inhomogeneous structure and/or irregular borders were considered suspicious for tumour invasion.

### Statistical analysis

The R software package (R version 3.1.3, The R Foundation for Statistical Computing; http:\\www.R-project.org\) was used for statistical calculation and obtaining data plots. The significance level for all calculations was set at P = 0.05. The Fisher-Exact test and the unpaired t test were used to test categorical or quantitative variables. Uni- and multivariate analyses were computed by using the Cox proportional hazards models. The following variables were considered for univariate analysis: patient age >70 years, neo- and adjuvant treatment, T3/4 versus T1/2 tumours, N1 versus N0, R1 versus R0, G3/4 versus G1/2, and PALN+ versus PALN−. The lymph node ratio (LNR) was calculated as the quotient between tumour infiltrated lymph nodes and resected lymph nodes, and was stratified in LNR <0.2 and ≥0.2. Significant variables on univariate analysis were entered into the multivariable test. The Kaplan–Meier method was used for overall and disease-free survival (DFS) curves, and the log-rank test was used to identify differences between the survival curves. OS was computed by the date of death or the time of last contact (censored), and DFS was defined as the time between the index operation and the last follow-up contact without disease progression. The follow-up time was defined from surgery to death, or the last patient contact.
